# Genome-wide identification and analysis of miRNA-related single nucleotide polymorphisms (SNPs) in rice

**DOI:** 10.1186/1939-8433-6-10

**Published:** 2013-04-23

**Authors:** Qingpo Liu, Hong Wang, Leyi Zhu, Haichao Hu, Yuqiang Sun

**Affiliations:** College of Agriculture and Food Science, Zhejiang A & F University, Lin’an Hangzhou, 311300 China; College of Life and Environmental Science, Hangzhou Normal University, Hangzhou, 310036 China

**Keywords:** Rice, SNP, miRNA, Structure stability, Target spectrum, Evolution

## Abstract

**Background:**

MiRNAs are key regulators in the miRNA-mediated regulatory networks. Single nucleotide polymorphisms (SNPs) that occur at miRNA-related regions may cause serious phenotype changes. To gain new insights into the evolution of miRNAs after SNP variation, we performed a genome-wide scan of miRNA-related SNPs, and analyzed their effects on the stability of miRNAs structure and the alteration of target spectrum in rice.

**Results:**

We find that the SNP density in pre-miRNAs is significantly higher than that in the flanking regions, owing to the rapid evolution of a large number of species-specific miRNAs in rice. In contrast, it is obvious that deeply conserved miRNAs are under strong purifying selection during evolution. In most cases, the SNPs in stem regions may result in the miRNA hairpin structures changing from stable to unstable status; And SNPs in mature miRNAs have great potential to have either newly created or disrupted the miRNA-target interactions. However, the total number of gained targets is over 2.5 times greater than that are lost after mutation. Notably, 12 putative domestication-related miRNAs have been identified, where the SNP density is significantly lower.

**Conclusions:**

The present study provides the first outline of SNP variations occurred in rice pre-miRNAs at the whole genome-wide level. These analyses may deepen our understanding on the effects of SNPs on the evolution of miRNAs in the rice genome.

**Electronic supplementary material:**

The online version of this article (doi:10.1186/1939-8433-6-10) contains supplementary material, which is available to authorized users.

## Background

Single nucleotide polymorphisms (SNPs) known as single-based differences among individuals within intra-species, are ubiquitously and abundantly present in most organisms (Arai-Kichise et al. 2011). As an important sequence variation, SNPs are developed as a type of DNA molecular marker, and widely used in genetic research including genome mapping, marker-assisted breeding, quantitative trait locus (QTL) analysis, and genome association analysis (Rafalski 2002; Jena and Mackill 2008; Lee et al. 2008; Huang et al. 2012a). To date, by virtual of the next generation sequencing technology, massive numbers of SNPs have been identified in animals and plants, including human (Lee et al. 2008), rice (Huang et al. 2012a; Xu et al. 2012 (Atwell et al. 2010), *Arabidopsis*), sorghum (Nelson et al. 2011), wheat (Lai et al. 2012), and etc. Genome-wide analyses show that SNPs are mainly dispersed in sequence regions with less conservation, such as intronic and intergenic regions, but fewer SNPs in functional regions, such as coding sequences (CDSs) and regulatory elements (Castle 2011). In most cases, functional SNPs can cause apparent phenotype changes or trait variations (Shastry 2009). For example, a single C-to-G point mutation in a non-coding RNA, osa-smR5864w, is a major determinant in controlling the pollen fertility or sterility in rice (Zhou et al. 2012). In common wheat, an A/G transition occurred at the nucleotide site 458 in CDS of *TaMYB2* gene is strongly associated with the dehydration tolerance of different cultivars (Garg et al. 2012).

Recently, several studies have extensively identified and analyzed the SNPs by re-sequencing the whole-genome of tens or hundreds of cultivated and/or wild plants (Atwell et al. 2010; Nelson et al. 2011; Huang et al. 2012a; Xu et al. 2012). However, most of these analyses focus on SNPs in protein-coding genes (Xu et al. 2012), and only a few studies mentioned the SNPs in miRNAs and target sites and their effects (Meng et al. 2011a; Wang et al. 2012a).

MiRNAs (microRNAs) are a class of endogenous small non-coding RNAs (Chen 2005). In plants, most miRNA genes that are usually very long can be transcribed by RNA polymerase II to give the primary miRNAs (pri-miRNAs) which are then processed into the precursor miRNAs (pre-miRNAs) by DCL1 in nucleus (Sun 2012). Pre-miRNAs can be further cleaved by DCL1 and HYL1 into a miRNA:miRNA* duplex (Kurihara et al. 2006), which is subsequently transported from nucleus into cytoplasm by HASTY (Park et al. 2005). In the cytoplasm, the miRNA:miRNA* duplex is separated, and the mature miRNA is incorporated into the RNA-induced silencing complex (RISC) to mediate the cleavage of target mRNAs (Chen 2005). Growing evidence shows that miRNAs are key regulators of gene expression, and play crucial roles in a diverse of biological processes (Bartel 2004; Chen 2005; Gielen et al. 2012; Sun 2012). Therefore, miRNA-related SNPs, especially SNPs in mature miRNAs and target sites may cause complex influence or severe consequences through modifying miRNA regulation. Hung et al. (2012) reported that a G-to-C polymorphism within pre-miR146a is significantly associated with the risk and occurrence of oral squamous cell carcinoma in human. In rice, *SPL14* is the target of osa-miR156, where an SNP occurred in the target site perturbs the normal miR156-*SPL14* interaction, and thereby leading to producing improved plant architecture (Jiao et al. 2010). In addition, SNPs in miRNAs can also affect the miRNA biogenesis and function. Sun et al. (2009) revealed that sequence variations in mature miRNAs and around the processing sites have profound influence on the mature miRNAs generation, processing and functional strand selection.

By whole-genome re-sequencing of 950 worldwide cultivars and 50 cultivars and wild accessions, Huang et al. (2012a) and Xu et al. (2012) have identified more than 4.1 and 6.5 millions high quality SNPs in rice, respectively. The high-throughput SNPs discovery made the genome-wide exploration of miRNA-related SNPs possible and feasible in rice. Since single nucleotide mutations occurred within the stem regions as well as mature miRNAs are important evolutionary powers for driving the production of new miRNAs by altering their biological functions (Sun et al. 2009), it is very intriguing to further uncover the SNPs characteristics and their possible effects on miRNAs evolution in the rice genome. Currently, there are 21,264 miRNAs deposited in miRBase (release 19, August 2012), of which 591 precursor sequences were derived from rice (; Kozomara and Griffiths-Jones 2011) *Oryza sativa*. Based on these populations of SNPs and miRNA data (pre-miRNAs), here we found that 364 out of the 591 rice miRNAs have one or more SNPs in their precursor sequences. The SNP density and their effects on miRNA structure stability and target alteration were further analyzed.

## Results

### Analysis of SNP characteristic in miRNA genes

**Figure 1:**
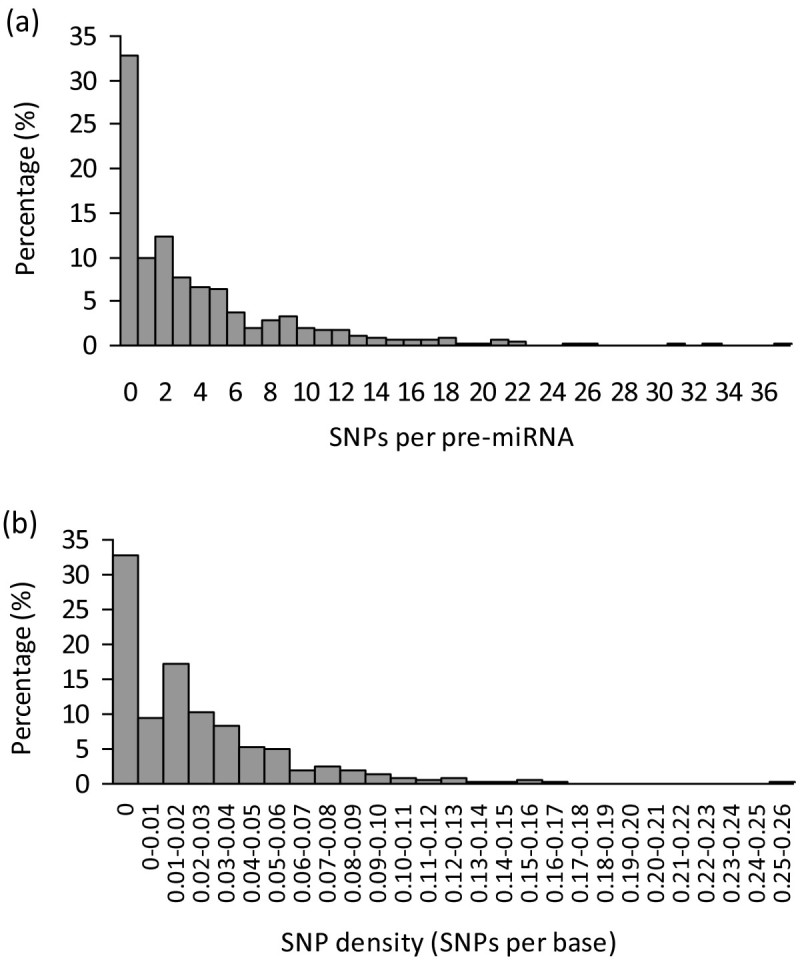
**Frequency distributions of different SNP numbers (a) and SNP density (b) in pre-miRNAs.**

Currently, a total of 591 precursor miRNAs were deposited in miRBase (release 19.0, Aug. 2012), of which 541 pre-miRNAs can be exactly mapped onto the IRGSP v4 rice genome. By comparing the 6.5 millions SNPs and 541 pre-miRNAs, 2,112 SNPs were identified in 364 pre-miRNAs, with an average of about 5.8 SNPs per pre-miRNA. Further analysis shows that 57.3% of pre-miRNAs have two or more SNPs (Figure 1), with 55 pre-miRNAs even having >10 SNPs in their precursor regions. Osa-miR2124b has the most SNPs (37), and followed by osa-miR812a (33) and osa-miR2124a (31). Notably, except for those ones with zero SNPs, pre-miRNAs having two SNPs are highly abundant, which accounts for 12.4% of total miRNA genes (541; Figure 1a). Moreover, the distribution of SNPs per base across the pre-miRNAs was analyzed as well. It is obvious that there are two peaks located in the 0 and 0.01-0.02 SNP density regions, which accounts for 32.7% and 17.2% of total miRNAs, respectively (Figure 1b).

**Figure 2:**
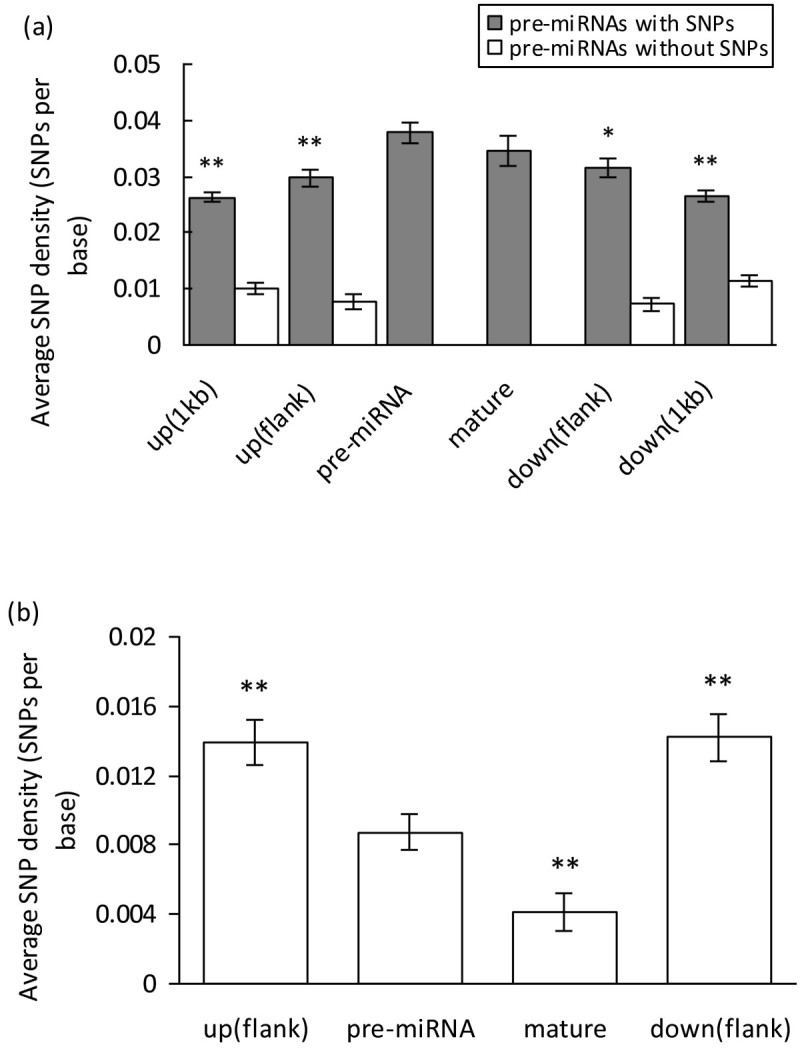
**Single nucleotide polymorphism (SNP) density of all rice pre-miRNAs (a), and deeply conserved miRNAs (b), and their flanking regions.** The up or down flank region represents a sequence region that is equal to the length of corresponding pre-miRNA, and located immediately adjacent to the pre-miRNA. The differences of SNP density between pre-miRNA and flanking regions were assessed using the ANOVA analysis. Data are reported as the average SNP density value ± *s*.*e*. The symbols * and ** designate the significant difference of SNP density in other regions as compared to that in pre-miRNAs at the 0.05 and 0.01 level, respectively.

To compare the SNP densities among different regions, we further characterized SNPs in the flanking regions of pre-miRNAs. Unexpectedly, we found that SNP densities of pre-miRNAs and mature miRNAs (MmiRNAs) are significantly higher than that in flanking regions, although the SNP density of MmiRNAs is lower than that in pre-miRNAs (Figure 2a). The further analyses of the 4.1 millions SNPs identified by Huang et al. (2012a) as well as the separate analysis of the cultivated (*indica*) and wild rice population SNPs (Xu et al. 2012) and *japonica* all gave the same trends (Additional file 1). It is worthy to note that the SNP density of pre-miRNAs has decreased and is lower than flanking regions, when the 55 pre-miRNAs with ≥10 SNPs were excluded (Additional file 2). On the contrary, as for the 177 pre-miRNAs without SNPs, the SNP densities in the up- and down-stream 1 kb regions are higher than that in the flanking regions immediately adjacent to the pre-miRNAs (Figure 2a). The similar phenomenon was also observed after assessing SNP densities based on the distances of different sequence regions to the mature miRNAs (Additional file 3).

In order to investigate whether the observed significant abundance of SNPs in pre-miRNAs and MmiRNAs are just caused by the integration of a large number of non-conserved miRNAs, the 541 rice pre-miRNAs were used to search against all the 24,433 mature miRNAs of other species deposited in miRBase (release 19.0) using BLASTN with E-value 10^-5^. Here, if a given miRNA has exact matches in other species, it is considered as evolutionarily conserved. Totally, 153 highly conserved pre-miRNAs were identified accordingly. Based on this dataset, significantly lower and higher SNP densities were observed in MmiRNAs and the two flanking regions, relative to the pre-miRNA region, respectively (Figure 2b). These results indicate that some miRNA genes should be under strong negative selection, whereas some others might evolve rapidly during evolution in rice.

**Figure 3:**
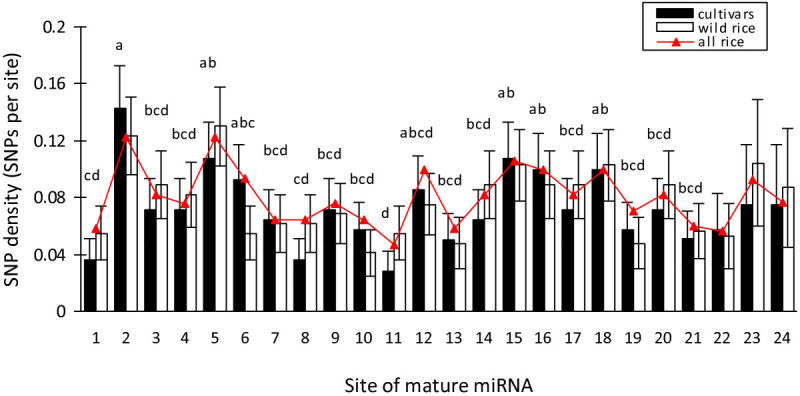
**SNP density for each site in rice mature miRNA regions.** Data are reported as the average SNP density value ± *s*.*e*. The different letters (***a*** **,** ***b*** **,** ***c***, and ***d***) designate the significant difference of SNP density between different sites at the 0.05 level.

Of the 2,112 SNPs identified, 307 are in 171 MmiRNAs. However, 56.1% and 23.4% MmiRNAs have only one or two SNPs, respectively. The analysis of distribution of SNPs per base along the MmiRNAs shows that 56.4% of MmiRNAs have an SNP density value ranging from 0.04 to 0.05 (Additional file 4). Moreover, by analyzing both the SNP numbers and SNP densities (SNPs per base) in MmiRNAs having different sequence length, we find that SNPs are prone to over-present in the ones with 22 nt in length, and followed by 24, and 21 nt (Additional file 4), although most MmiRNAs are 21, 22, or 24 nt in length. We subsequently calculated and compared the SNP density for each site in MmiRNAs, and found that three nucleotide sites including 1, 8, and 11 tend to have accumulated significantly fewer SNPs than the sites 2, 5, 15, 16, and 18 in cultivated rice (Figure 3).

**Figure 4:**
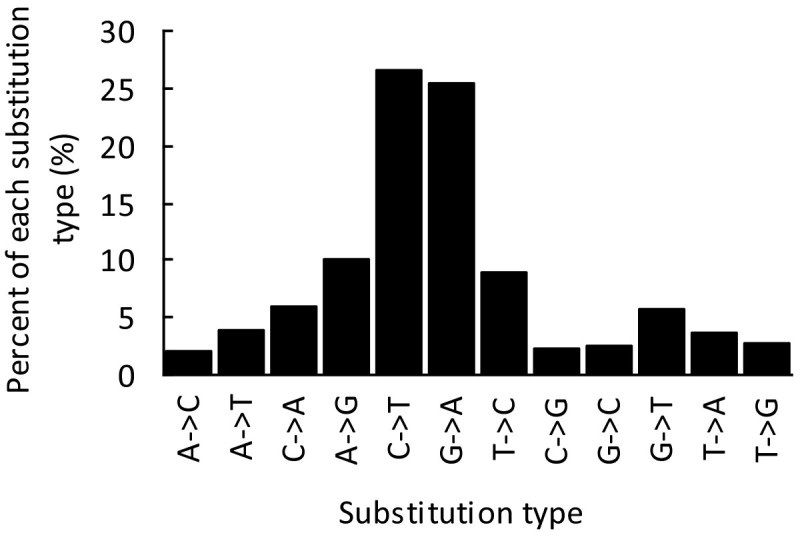
**Illustration of substitution types for SNPs that occur in rice pre-miRNAs.** Arrows indicate the direction of single nucleotide mutation. The ancestral state of each SNP site was derived from the Rice Haplotype Map Project Database (Huang et al. 2012b).

In addition, by analyzing the ancestral state of each SNP site from the Rice Haplotype Map Project Database (Huang et al. 2012b), we found that transitions (Tr; 71.1%) were far more frequent than transversions (Tv; 28.9%) in rice pre-miRNAs (Tr/Tv = 2.46). The most frequent substitutions are C-to-T (26.6%) and G-to-A (25.4%; Figure 4 small RNA sequencing data (Ebhardt et al.^2009^), which is similar to the results obtained by using the rice and *Arabidopsis*. Coulondre et al. (1978) showed that true SNPs are abundantly biased to transitions rather than transversions. Thus, the observed transition abundance bias may reflect a higher level of methylation in pre-miRNAs in the rice genome (Coulondre et al. 1978).

### Effects of SNPs on the stability of miRNAs secondary structure

**Figure 5:**
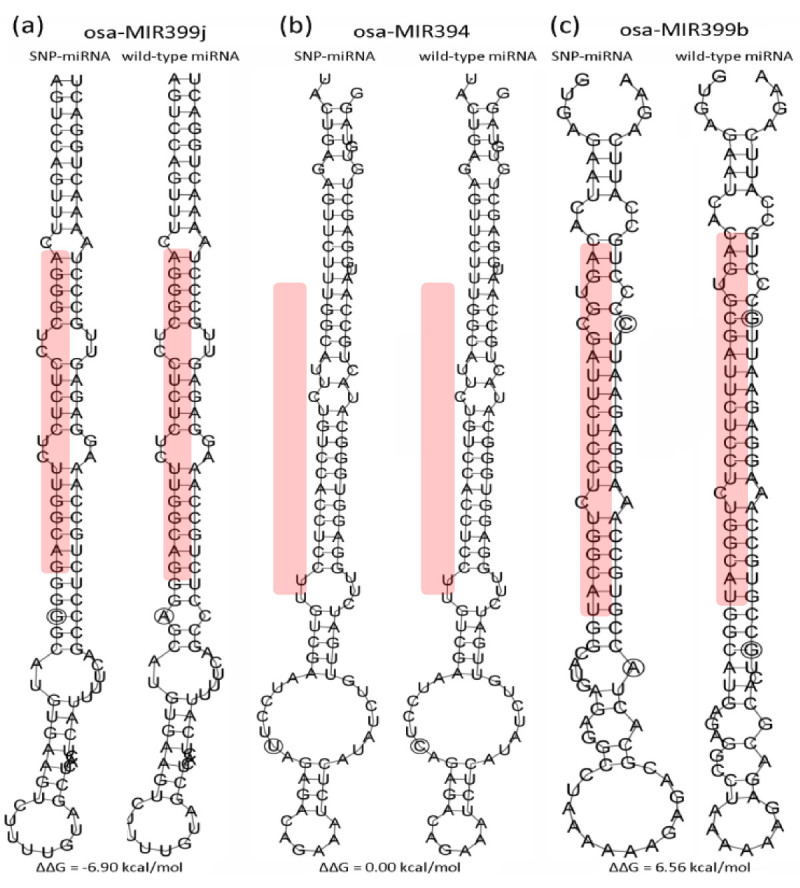
**Illustration of the predicted secondary structures for SNP- and wild-type osa-miR399j (a), osa-miR394 (b), and osa-miR399b (c).** The mature miRNAs are shaded in pink. The circle marks the SNP position. The three miRNAs are shown as typical examples for displaying the increased (ΔΔG = −6.90 kcal/mol), unchangeable (ΔΔG = 0.00 kcal/mol), and decreased stability of hairpin structures (ΔΔG = 6.56 kcal/mol) by SNP variations.

**Figure 6:**
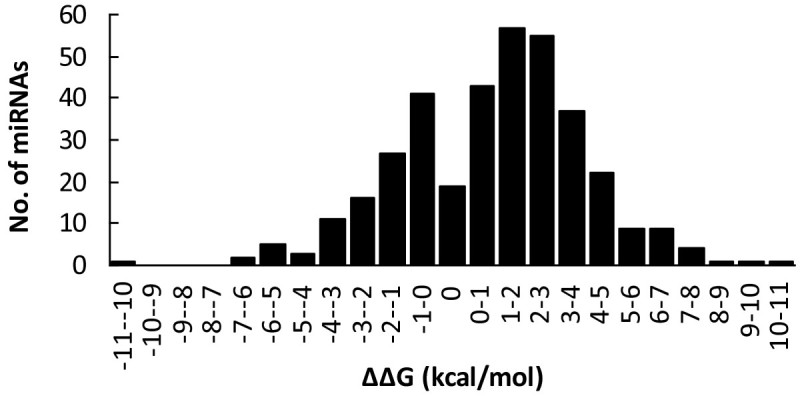
**Histogram displaying the distribution of energy change (ΔΔG, kcal/mol) of miRNA hairpin structures caused by SNPs in rice pre-miRNAs.**

In order to investigate the influence of SNPs in pre-miRNAs on the stability of hairpin structure, we used the RNAfold program (Hofacker 2003) to predict the secondary structures (Figure 5), and compare the energy change (ΔΔG) caused by SNPs for the wild- and SNP-type pre-miRNAs. It was observed that 63.7% of miRNAs (232 out of 364) have changed their hairpin structures from stable to unstable status, such as from A:U to C:U (Figure 5c); their energy change (ΔΔG) values are between 0.3 to 10.1 kcal/mol, which are greater than the reported minimum energy change required for affecting the generation of mature miRNAs (Sun et al. 2009). For nineteen miRNAs that belong to 15 miRNA families, such as osa-miR2101, osa-miR171a and h, osa-miR169l and q, the SNPs mainly occurred in the loop regions (Figure 5b), and thereby leading to no obvious energy changes in their hairpin structures. On the other hand, the SNPs in 99 pre-miRNAs (27.2%) increased their hairpin stability, with an energy change from −10.0 to −0.3, respectively. A typical example is the osa-miR399j, where an A-to-G SNP in the stem region made its hairpin structure much stable (A:C to G:C; Figure 5a). In addition, there are 13 pre-miRNAs having the energy changes from −0.2 to −0.1, or from 0.1 to 0.2, respectively. Further analysis showed that the slightly change of the stability of these hairpin structures is mainly due to the changes from A:U to G:U, or vice versa. Overall, 48.6% of miRNAs are found to have an energy change (|ΔΔG|) ≥2.0 kcal/mol (Figure 6). In average, the absolute energy change of pre-miRNA secondary structures (|ΔΔG|) caused by SNP is 2.41 kcal/mol in rice, which is higher than that found in the humans genome (2.1 kcal/mol; Gong et al. 2012).

### Target spectrum alteration by SNPs in mature miRNAs

In plants, miRNAs can form perfect or near perfect complementary hybrids to targets, and hence guiding the cleavage of target mRNAs (Voinnet 2009). Thus, if an SNP occurs at the mature miRNA, it may cause mismatches between miRNA and target binding sites, and further affects the miRNA function. As mentioned above, we have identified 307 SNPs within the mature sequences of 171 miRNAs in rice. With the exceptions of the 171 wild-type miRNAs (WmiRNAs), a total of 312 new miRNAs (SNP-miRNAs; SmiRNAs) have been obtained after projecting the SNPs onto the miRNAs (Additional file 5). Further analysis showed that 57.3% (98 out of 171) of SNP-miRNAs have produced only one new mature miRNAs each, whereas the remaining miRNAs have two or more new variations, with the osa-miR2124h being the most (8) (Additional file 5).

**Figure 7:**
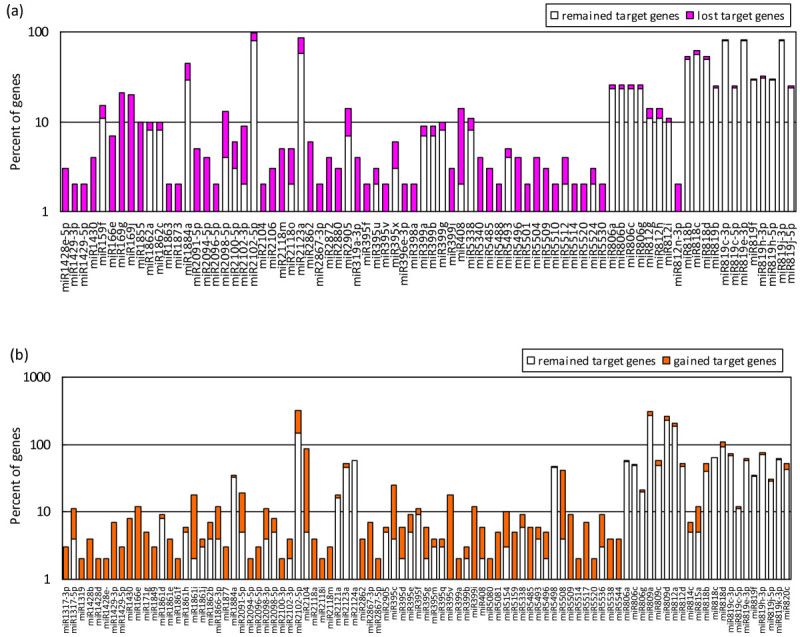
**Target spectrum alteration analyses of miRNAs by SNPs in their mature sequence regions.** The number of targets that were lost (**a**) or gained (**b**) was presented by comparison of wild-type miRNAs with SNP-miRNAs. To facilitate the comparison, the Y-axis is shown in log (10) scale.

Using a combination of two plant miRNA target prediction methods, the target spectrum of WmiRNAs and SmiRNAs were analyzed. In total, 2,576 and 2,914 potential targets for WmiRNAs, and 4,310 and 4,869 for SmiRNAs were predicted by PsRobot (Wu et al. 2012) and Target_prediction (Sun et al. 2010), respectively. Finally, there are 1,889 and 3,179 potential targets predicted by both methods for the wild- (171) and SNP-miRNAs (312), respectively. By comparing the targets of WmiRNA with that of SmiRNA, we found that 736 potential target genes for 91 miRNAs, with an average of 8.1 targets per miRNA, were newly created, while 287 targets for 78 miRNAs were disrupted by SNPs variation (Figure 7). Three representative examples were listed in Additional file 6 to show the alignments of WmiRNA and SmiRNA to the predicted target mRNAs. By analyzing the relationships between miRNAs and the lost/gained target genes, we find that some miRNAs, such as osa-miR2102-5p osa-miR2104, and osa-miR5508, have gained 175, 82, and 38 potential targets after variation, respectively. Notably, osa-miR2104 and osa-miR5508 have gained more than 94% and 90% of target genes. In contrast, all the putative targets of osa-miR169g and osa-miR169j have been lost after mutation. In addition, some osa-miRNAs have no but gained one or several targets, *e*.*g*. osa-miR2862, osa-miR2867-3p, while some miRNAs, such as osa-miR1873, osa-miR2094-5p, have only several putative targets but was lost completely after variation (Figure 7).

### Analysis of SNP characteristic of miRNAs located in the putative domestication-related regions

Using the high-throughput sequencing approach, Xu et al. (2012) identified 739 and 750 putative domestication-related sequence regions in *japonica* and *indica* rice, respectively. Based on these regions, 29 putative domestication-associated miRNA genes were characterized. In addition, 24, 36, and 50 miRNAs were identified from other studies by searching the sequence regions with extremely low level of polymorphisms in cultivated but not in wild rice (He et al. 2011; Yang et al. 2012; Yonemaru et al. 2012). Taken together, here, if a miRNA was found within three out of the four studies mentioned above, it was considered as a putative domestication-related candidate (DR-miRNA). Totally, 12 putative DR-miRNAs, including osa-miR5513, osa-miR818e, osa-miR5158, osa-miR1847, osa-miR1865, osa-miR160f, osa-miR5143, and osa-miR2118h-l, were used for further analysis.

**Figure 8:**
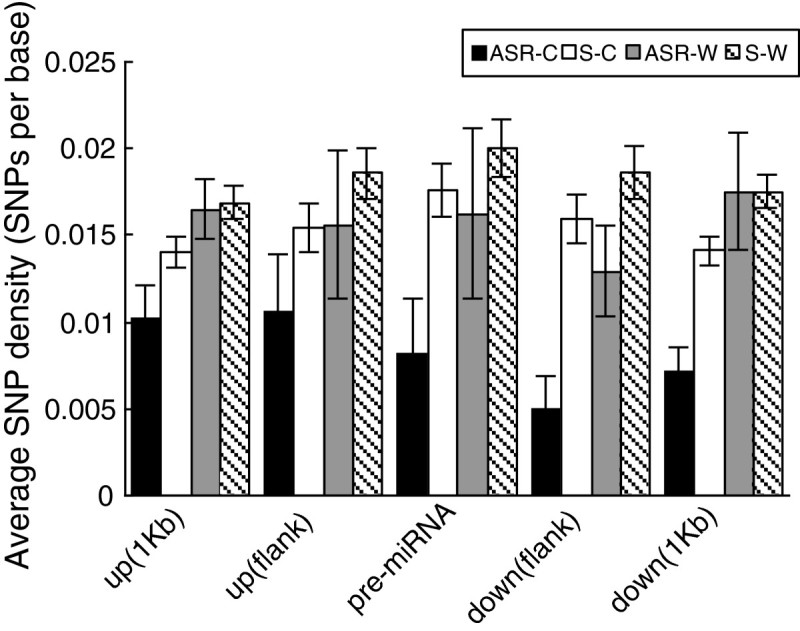
**Comparison of SNP density in the precursor sequences (pre-miRNAs) and flanking regions for putative domestication-related miRNAs (DR-miRNAs) and other miRNAs in cultivated and wild rice.** For comparison, 2,000 independent permutations were performed by randomly choosing 12 miRNAs from the cultivars and wild rice miRNA datasets, respectively. Data are reported as the average SNP density value ± *s*.*e*. *ASR-C* and *ASR-W* mean DR-miRNAs in cultivated and wild rice, respectively; *S-C* and *S-W* designate other miRNAs (12 miRNAs per permutation) randomly extracted from cultivars and wild rice, respectively.

As shown in Figure 8, the SNP density, either in the pre-miRNAs or flanking regions, is significantly lower in cultivars as compared to wild rice. In addition, the SNP density in the immediately down-stream flanking region is even significantly lower than that of the pre-DR-miRNAs (Figure 8). In order to test whether the observed low sequence diversity in pre-DR-miRNAs and their immediately down-stream regions was caused just by chance in cultivars, 2,000 independent permutations were performed by randomly extracting 12 miRNAs from the cultivar and wild rice miRNA datasets, respectively, and the SNP densities for the pre-miRNAs and flanking regions were re-calculated accordingly (Figure 8). It is found that DR-miRNAs have the fewest SNPs compared to other three datasets. These results indicate that DR-miRNAs are indeed to have significantly fewer SNPs than other miRNAs that depends on the sequence regions where they are located.

## Discussion

A line of evidence shows that single nucleotide polymorphisms (SNPs) are important functional sequence variations, and SNPs that occur at miRNA loci may have profound effects during the evolution of a species. To date, a survey of the concerted evolution of miRNAs and their binding target sites has been investigated in *Arabidopsis* (Ehrenreich and Purugganan 2008), and rice (Wang et al. 2010), respectively. In these analyses, a significant lower level of polymorphism in miRNAs was found, indicative of purifying selection in these pre-miRNAs (Ehrenreich and Purugganan 2008; Wang et al. 2010); while the reverse phenomenon was observed in this study when all rice miRNA genes were taken into consideration, where pre-miRNAs accumulated more SNPs than the flanking regions (Figure 2a). The major reason underlying the observed inconsistence may be due to the differences in evolutionary conservation of the selected miRNA samples in different studies, because when only the highly conserved rice pre-miRNAs were adopted, the SNP densities in pre-miRNAs and MmiRNAs decreased significantly (Figure 2b). As known, the miRNA repertoire of each species basically consists of a set of conserved miRNAs as well as many lineage- or species-specific miRNAs (Rajagopalan et al. 2006). During evolution, significantly stronger selective constraints should impose on the deeply conserved miRNAs, whereas most species-specific miRNAs (young miRNAs) are expressed weakly, or in specific conditions or tissue types, more divergent, and tend to lack targets, suggesting a neutrally evolving path. For example, we analyzed further the 55 pre-miRNAs with 10 or more SNPs, particularly the miR2124 family members (osa-miR2124a-f, h-i), and found that these miRNAs are almost rice species-specific. However, using a very stringent filter approach, we found that 44 out of the 177 pre-miRNAs having no SNPs including osa-miR156e-j, osa-miR160a, osa-miR166b, f, osa-miR172a, c, d, osa-miR395j, l, and etc. are highly conserved in plants. These results support the findings in human that species-specific miRNAs that are always lowly expressed are under weaker selective pressures and evolve rapidly (Liang and Li 2009).

In rice, the length of mature miRNAs (MmiRNAs) ranges from 19 to 24 nt, where near half (49.2%) are 21 nt in length, and followed by 22 nt (22.2%) and 24 nt (20.3%). However, 28.7%, 36.5% and 31.9% SNPs were found to be located in the MmiRNAs with 21, 22, and 24 nt in length, respectively. This observation suggests that weaker constraints should work on the 22- and 24-nt-MmiRNAs, leading to the generation of new miRNAs or alteration their functions. In plants, most mature miRNAs start with uridine (U; site 1); sites 10, 11, and 19 are crucial for target recognition and cleavage (Palatnik et al. 2007; Voinnet 2009), which explains the low sequence diversity at these sites (Figure 3). In addition, the nucleotide site 8 is also rather conserved during evolution, suggestive of its potential importance in miRNA-target interactions and/or functions.

Several studies revealed that SNPs in miRNA genes could affect, either increase or disrupt the processing of miRNAs. However, most of these analyses have been performed in human (Sun et al. 2009; Harnprasopwat et al. 2010; Gong et al. 2012). For example, an A/G SNP that is located at the 24-nucleotide downstream of the 3’ end of the mature miR126, can significantly block the processing of pri-miR126 to mature miRNA in the RS4;11 cells (Harnprasopwat et al. 2010). On the contrary, a G/A transition occurring in the stem region of miR510 enhanced the production of mature miRNA (Sun et al. 2009). By analyzing the published documents (Jazdzewski et al. 2008; Sun et al. 2009; Harnprasopwat et al. 2010) as well as large-scale analysis of human pre-miRNAs SNPs, Gong et al. (2012) summarized a possible relationship between the SNPs in miRNAs stem and their effects on the generation of mature miRNAs. Based on this rule, we can infer that most of rice miRNAs would reduce the mature miRNAs production, thanks to the instability of hairpin structures caused by SNPs in stem regions. In contrast, near one third of miRNAs could increase the efficiency of processing pre-miRNAs into mature miRNAs, because the SNPs in stems extensively enhanced the stability of hairpin structures.

As reported, miRNAs can not only down-regulate the expression of proteins at the translational level (Brodersen et al. 2008), but also efficiently guide the cleavage of genes mRNA at the post-transcriptional level (Voinnet 2009). It is estimated that approximately 30% of human mRNA genes may be regulated by miRNAs (Friedman et al. 2009). In plants, the total number of genes targeted by miRNAs is greatly decreased compared with that in animals (Jones-Rhoades and Bartel 2004). Accordingly, plant miRNAs have fewer targets relative to animal miRNAs. For example, most *Arabidopsis* miRNAs have on average six targets or fewer (Jones-Rhoades et al. 2006), whereas each human miRNA can target hundreds of genes (Gong et al. 2012). Here, we found that each WmiRNA has on average 11 targets in rice, which is significantly fewer than that in human (375 targets per wild-type miRNA; Gong et al. 2012). Interestingly, increasing evidence reveals that miRNAs constitute a large and complex gene regulatory network in plants (Meng et al. 2011b). In most cases, one miRNA can target many different mRNA genes; In contrast, one mRNA can also be regulated by two or more miRNAs. We found for example that both the osa-miR820c and osa-miR395 family members can co-target the *LOC_Os03g53230.1*, a gene that encodes bifunctional 3-phosphoadenosine 5-phosphosulfate synthetase that is highly abundant in the roots of 7-day-old rice seedlings (GSE6893). Two F-box domain-containing protein genes, *LOC_Os12g32630.1* and *LOC_Os12g32630.2*, are the newly created targets of SNP-miR2104 and SNP-miR2102-5p, suggestive of the gain of additional roles for the two SmiRNAs in development transition and in response to environmental stressors in plants (Jain et al. 2007).

It is generally accepted that the modern rice (*Oryza sativa*) has been domesticated from its Asian wild ancestor *O. rufipogon* about 10,000 years ago (Kovach et al. 2007). After re-sequencing tens to hundreds of rice genotypes, Huang et al. (2012b) and Xu et al. (2012) demonstrated that artificial selection should impose on the whole rice genome during the long period time of domestication. During the past decades, more than 11 protein-coding genes controlling domestication traits have been identified in rice (Izawa et al. 2009; Jiao et al. 2010). However, the issue whether miRNA genes were under artificial selection during domestication remains to be further clarified. In this study, twelve putative domestication-related miRNAs were identified by searching and comparing the whole rice genome sequences. In the previous studies, Ehrenreich and Purugganan (2008) and Wang et al. (2007; 2010) found evidence for positive selection on some miRNA loci in *Arabidopsis* and rice, respectively. Notably, the investigation of the whole miRNA repertoire of cultivated and wild rice (*O. rufipogon*) has supported the notion that some miRNA genes should have experienced strong artificial selection during evolution (Wang et al. 2012b). Therefore, miRNA genes, like protein-coding genes, could serve as one of the driving forces that are important for rice domestication (Wang et al. 2012b).

## Conclusions

Based on the identified several millions of high-quality SNPs in rice, a genome-wide scan of SNPs in pre-miRNAs was performed. We find that compared with deeply conserved miRNAs, young miRNAs tend to have accumulated significantly higher SNPs in their precursor sequences. SNPs occurred at the stem regions and mature miRNAs (MmiRNAs) may extensively change the stability of miRNAs secondary structures and the MmiRNA-target interactions, respectively, and thereby further affecting the miRNAs biogenesis and potential functions. In addition, the significantly lower SNP density in the putative domestication-related miRNAs may be suggestive of their experience of stronger artificial or natural selection. These findings are important to better understand how miRNAs are evolved under natural selection and domestication during evolution of the rice genome.

## Methods

### Sequence data

The rice miRNA data including the precursor sequences and mature miRNAs were derived from the miRBase database (release 19.0). The 4.1 and 6.5 millions high quality SNPs were downloaded from the Rice Haplotype Map Project Database (http://202.127.18.221/RiceHap2/index.php) and the BGI rice database (ftp://rice:ricedownload@public.genomics.org.cn/BGI/rice), respectively. The two SNP datasets were all based on the IRGSP v4 rice genomic sequences (Huang et al. 2012a; Xu et al. 2012).

### Identification of miRNA-related SNPs

The 591 rice pre-miRNAs were used as query to search against the IRGSP v4 genomic sequences using BLASTN with E-value cutoff 10^-10^. If a given pre-miRNA sequence was perfectly mapped to the IRGSP v4 rice genome, its chromosomal coordinate information was extracted using in-house PERL programs. Then, the chromosomal locations of pre-miRNAs and SNPs were compared to identify SNPs that are located in mature miRNAs, precursor sequences, and the proximate up- and down-stream regions. The SNP density that is assessed by the SNP numbers per base was calculated and compared for pre-miRNAs and flanking regions. The differences in SNP density between pre-miRNAs and other sequence regions were evaluated using the ANOVA analysis. The program RNAfold was employed to generate the pre-miRNA hairpin structures, and to calculate the minimum free energies (Hofacker 2003). The secondary structures of pre-miRNAs were displayed by RNAplot (Hofacker 2003).

### Target alteration analysis

For the miRNA genes with SNPs in their mature miRNAs, two plant miRNA target prediction tools target_prediction (Sun et al. 2010) and PsRobot (Wu et al. 2012) with the default parameters were utilized to analyze the target gain and loss events for the wild-type (WmiRNAs) and SNP-type miRNAs (SmiRNAs). Briefly, if one miRNA/target pair was found by both the two methods for the WmiRNA but not for the SmiRNA, we defined that the SmiRNA lost the target gene. In contrast, we called that the SmiRNA gained the corresponding target, if one mRNA gene was predicted to be the target for the SmiRNA but not for the WmiRNA by both methods (Gong et al. 2012).

## Electronic supplementary material

Additional file 1: Figure S1: SNP density of rice pre-miRNAs and flanking regions, based on the Huang et al. (2012) SNP data (a), and the Xu et al. (2012) SNPs where the cultivar and wild rice SNP populations were separately analyzed (b). The up or down flank region represents a sequence region that is equal to the length of corresponding pre-miRNA, and located immediately adjacent to the pre-miRNA. Data are reported as the average SNP density value ± *s*.*e*. (DOC 47 KB)

Additional file 2: Figure S2: SNP density of rice pre-miRNAs and flanking regions that was calculated based on the 309 miRNAs with ≤ 10 SNPs (a), and 55 miRNAs having 10 or more SNPs each in their precursor sequences (b). The up or down flank region represents a sequence region that is equal to the length of corresponding pre-miRNA, and located immediately adjacent to the pre-miRNA. Data are reported as the average SNP density value ± *s*.*e*. (DOC 42 KB)

Additional file 3: Figure S3: SNP density of each sequence region that is up- or down-stream of the mature miRNAs. In this analysis, an approach that is based on the distance to mature miRNAs is adopted. The average sequence length of pre-miRNAs with or without SNPs (159 and 150 nt, respectively) is used to calculate the SNP density for each sequence region. The up or down flank region represents a sequence region that is located adjacent to the mature miRNA. The differences of SNP density between sequence regions are assessed using the ANOVA analysis. Data are reported as the average SNP density value ± *s*.*e*. The different letters (*a*, *b*, *c*, and *d*) designate the significant difference of SNP density between different regions at the 0.05 level. (DOC 52 KB)

Additional file 4: Figure S4: Frequency distributions of mature miRNAs with different SNP numbers (a), different SNP density (b), and cumulative frequency distributions of SNPs and SNP density in mature miRNAs having different length (c-d). In panel d, data are reported as the average SNP density value ± *s*.*e*; The different letters (*a* and *b*) designate the significant difference of SNP density between different regions at the 0.05 level. (DOC 46 KB)

Additional file 5: Table S1: List of wild-type and SNP-type mature miRNAs. (XLS 76 KB)

Additional file 6: Table S2.: Sequence alignments of target genes with the wild- and SNP-type-miRNAs in rice. (XLS 28 KB)

Below are the links to the authors’ original submitted files for images.

Authors’ original file for figure 1

Authors’ original file for figure 2

Authors’ original file for figure 3

Authors’ original file for figure 4

Authors’ original file for figure 5

Authors’ original file for figure 6

Authors’ original file for figure 7

Authors’ original file for figure 8
