# Supplementary material for: Genome-wide identification and analysis of miRNA-related single nucleotide polymorphisms (SNPs) in rice
Source: Rice (N Y). 2013 Apr 23;6:10. doi: 10.1186/1939-8433-6-10 (PMC4883715; doi:10.1186/1939-8433-6-10)
Supplement: Supplementary file 1 — Additional file 1: Figure S1: SNP density of rice pre-miRNAs and flanking regions, based on the Huang et al. (2012) SNP data (a), and the Xu et al. (2012) SNPs where the cultivar and wild rice SNP populations were separately analyzed (b). The up or down flank region represents a sequence region that is equal to the length of corresponding pre-miRNA, and located immediately adjacent to the pre-miRNA. Data are reported as the average SNP density value ± s.e. (DOC 47 KB) [file 12284_2012_46_MOESM1_ESM.doc]

**Supplementary Figure 1**

SNP density of rice pre-miRNAs and flanking regions, based on the Huang et al. (2012) SNP data (a), and the Xu et al. (2012) SNPs where the cultivar and wild rice SNP populations were separately analyzed (b). The up or down flank region represents a sequence region that is equal to the length of corresponding pre-miRNA, and located immediately adjacent to the pre-miRNA. Data are reported as the average SNP density value ± *s*.*e*.
