# Supplementary material for: Genome-wide identification and analysis of miRNA-related single nucleotide polymorphisms (SNPs) in rice
Source: Rice (N Y). 2013 Apr 23;6:10. doi: 10.1186/1939-8433-6-10 (PMC4883715; doi:10.1186/1939-8433-6-10)
Supplement: Supplementary file 2 — Additional file 2: Figure S2: SNP density of rice pre-miRNAs and flanking regions that was calculated based on the 309 miRNAs with ≤ 10 SNPs (a), and 55 miRNAs having 10 or more SNPs each in their precursor sequences (b). The up or down flank region represents a sequence region that is equal to the length of corresponding pre-miRNA, and located immediately adjacent to the pre-miRNA. Data are reported as the average SNP density value ± s.e. (DOC 42 KB) [file 12284_2012_46_MOESM2_ESM.doc]

**Supplementary Figure 2**

SNP density of rice pre-miRNAs and flanking regions that was calculated based on the 309 miRNAs with ≤ 10 SNPs (a), and 55 miRNAs having 10 or more SNPs each in their precursor sequences (b). The up or down flank region represents a sequence region that is equal to the length of corresponding pre-miRNA, and located immediately adjacent to the pre-miRNA. Data are reported as the average SNP density value ± *s*.*e*.
