# Supplementary material for: Genome-wide identification and analysis of miRNA-related single nucleotide polymorphisms (SNPs) in rice
Source: Rice (N Y). 2013 Apr 23;6:10. doi: 10.1186/1939-8433-6-10 (PMC4883715; doi:10.1186/1939-8433-6-10)
Supplement: Supplementary file 3 — Additional file 3: Figure S3: SNP density of each sequence region that is up- or down-stream of the mature miRNAs. In this analysis, an approach that is based on the distance to mature miRNAs is adopted. The average sequence length of pre-miRNAs with or without SNPs (159 and 150 nt, respectively) is used to calculate the SNP density for each sequence region. The up or down flank region represents a sequence region that is located adjacent to the mature miRNA. The differences of SNP density between sequence regions are assessed using the ANOVA analysis. Data are reported as the average SNP density value ± s.e. The different letters (a, b, c, and d) designate the significant difference of SNP density between different regions at the 0.05 level. (DOC 52 KB) [file 12284_2012_46_MOESM3_ESM.doc]

**Supplementary Figure 3**

Single nucleotide polymorphism (SNP) density of each sequence region that is up- or down-stream of the mature miRNAs. In this analysis, an approach that is based on the distance to mature miRNAs is adopted. The average sequence length of pre-miRNAs with or without SNPs (159 and 150 nt) is used to calculate the SNP density for each sequence region. The up or down flank region represents a sequence region that is located adjacent to the mature miRNA. The differences of SNP density between sequence regions are assessed using the ANOVA analysis. Data are reported as the average SNP density value ± *s*.*e*. The different letters (*a*, *b*, *c*, and *d*) designate the significant difference of SNP density between different regions at the 0.05 level.
