# Supplementary material for: Genome-wide identification and analysis of miRNA-related single nucleotide polymorphisms (SNPs) in rice
Source: Rice (N Y). 2013 Apr 23;6:10. doi: 10.1186/1939-8433-6-10 (PMC4883715; doi:10.1186/1939-8433-6-10)
Supplement: Supplementary file 4 — Additional file 4: Figure S4: Frequency distributions of mature miRNAs with different SNP numbers (a), different SNP density (b), and cumulative frequency distributions of SNPs and SNP density in mature miRNAs having different length (c-d). In panel d, data are reported as the average SNP density value ± s.e; The different letters (a and b) designate the significant difference of SNP density between different regions at the 0.05 level. (DOC 46 KB) [file 12284_2012_46_MOESM4_ESM.doc]

**Supplementary Figure 4**

Frequency distributions of mature miRNAs with different SNP numbers (a), different SNP density (b), and cumulative frequency distributions of SNPs and SNP density in mature miRNAs having different length (c-d). In panel d, data are reported as the average SNP density value ± *s*.*e*; The different letters (*a* and *b*) designate the significant difference of SNP density between different regions at the 0.05 level.
