# Supplementary figures and images for: Genome-wide identification and analysis of miRNA-related single nucleotide polymorphisms (SNPs) in rice
Source: Rice (N Y). 2013 Apr 23;6:10. doi: 10.1186/1939-8433-6-10 (PMC4883715; doi:10.1186/1939-8433-6-10)

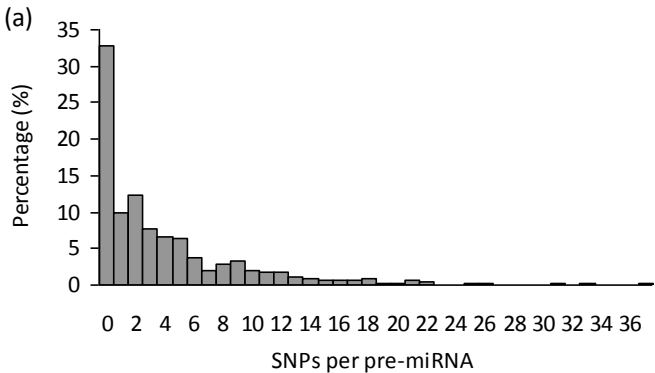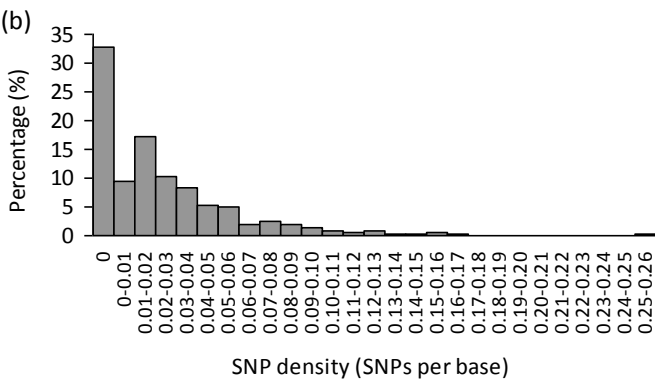

Supplement: Supplementary file 7 — Authors’ original file for figure 1 [file 12284_2012_46_MOESM7_ESM.pdf]

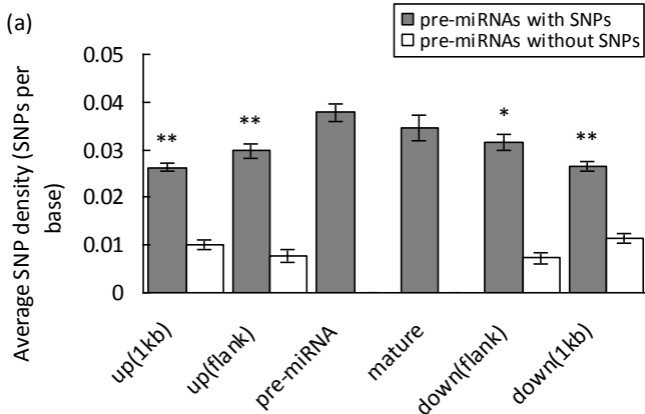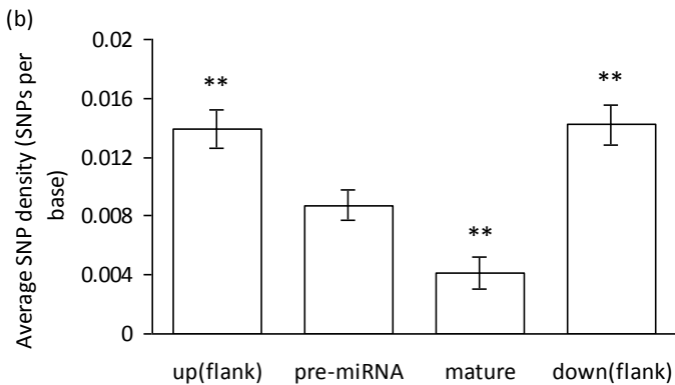

Supplement: Supplementary file 8 — Authors’ original file for figure 2 [file 12284_2012_46_MOESM8_ESM.pdf]

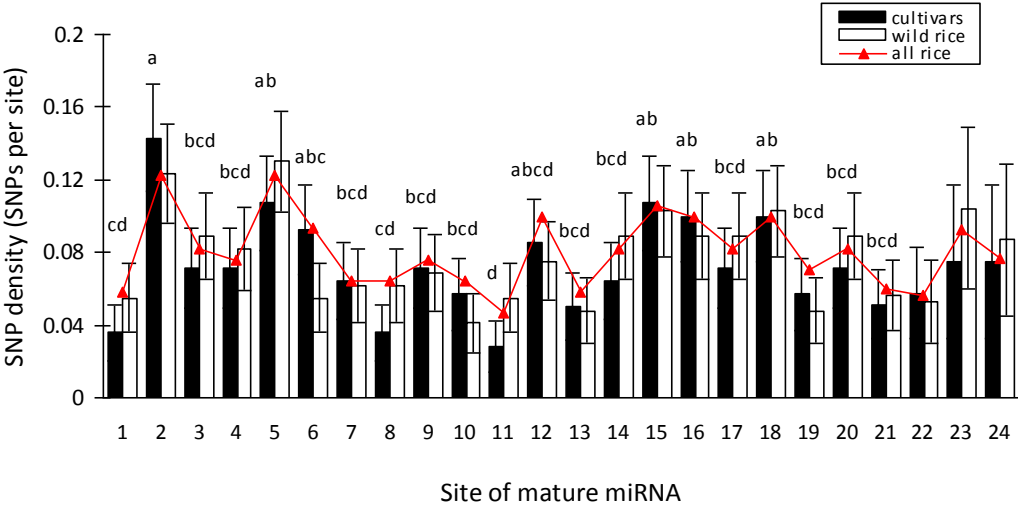

Supplement: Supplementary file 9 — Authors’ original file for figure 3 [file 12284_2012_46_MOESM9_ESM.pdf]

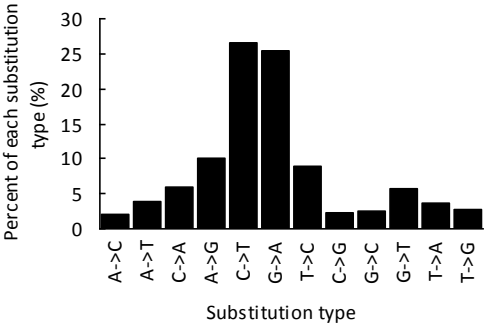

Supplement: Supplementary file 10 — Authors’ original file for figure 4 [file 12284_2012_46_MOESM10_ESM.pdf]

(c) osa-MIR399b

wild-type miRNA

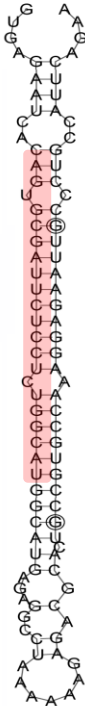
$$\Delta\Delta G = 6.56 \text{ kcal/mol}$$

Supplement: Supplementary file 11 — Authors’ original file for figure 5 [file 12284_2012_46_MOESM11_ESM.pdf]

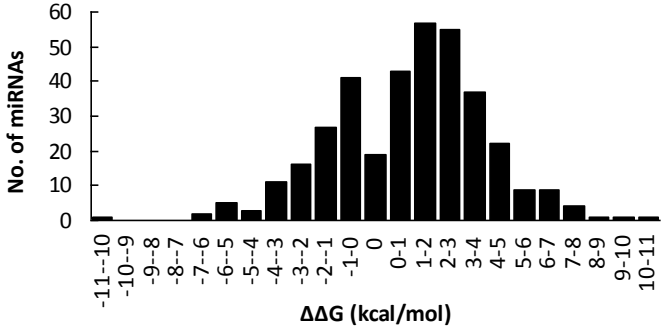

Supplement: Supplementary file 12 — Authors’ original file for figure 6 [file 12284_2012_46_MOESM12_ESM.pdf]

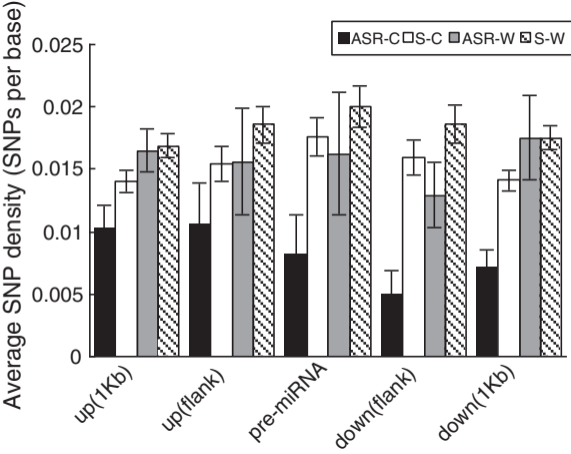

Supplement: Supplementary file 14 — Authors’ original file for figure 8 [file 12284_2012_46_MOESM14_ESM.pdf]
