# Supplementary material for: Genome-wide identification and analysis of miRNA-related single nucleotide polymorphisms (SNPs) in rice
Source: Rice (N Y). 2013 Apr 23;6:10. doi: 10.1186/1939-8433-6-10 (PMC4883715; doi:10.1186/1939-8433-6-10)
Supplement: Supplementary file 13 — Authors’ original file for figure 7 [file 12284_2012_46_MOESM13_ESM.pdf]

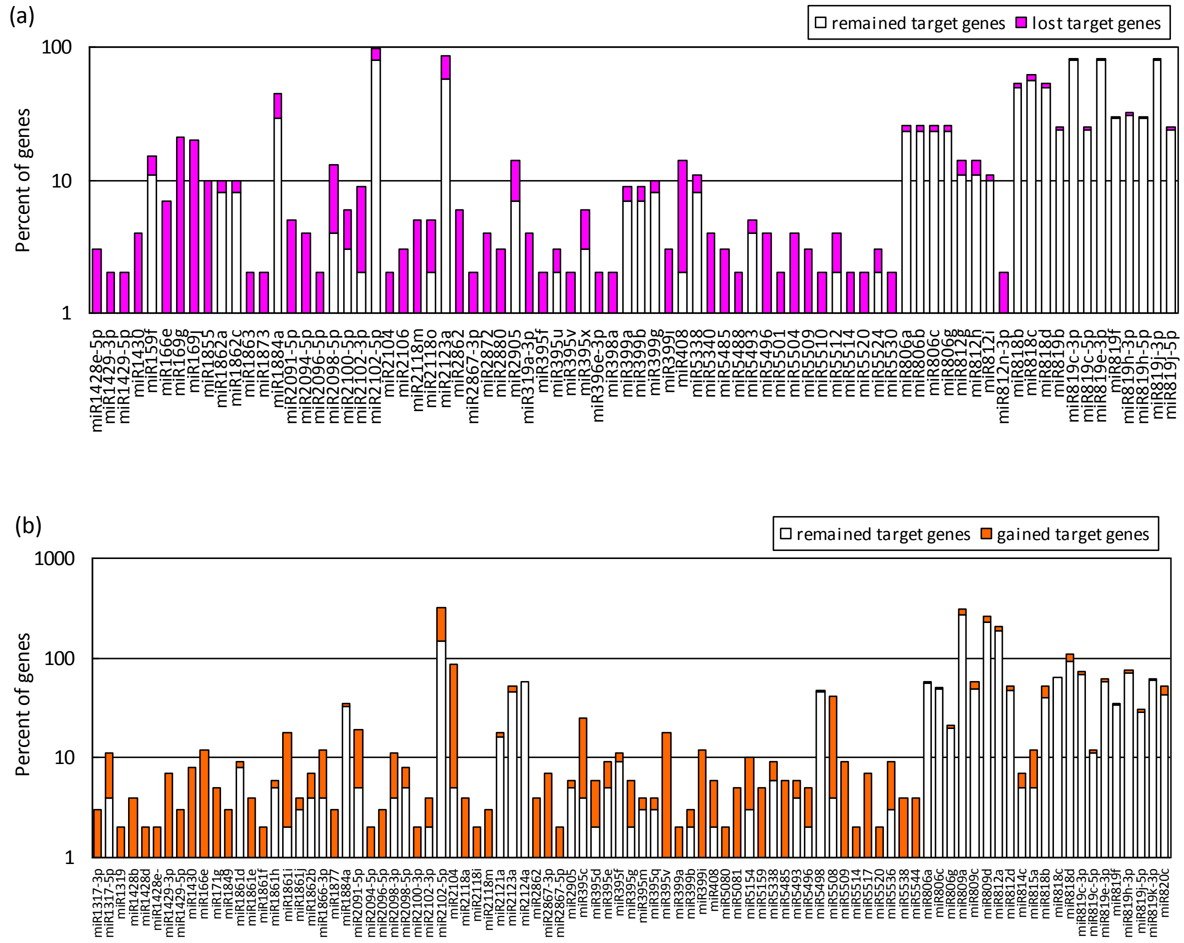

(a)
remained target genes
lost target genes
Percent of genes
100
10
1
miR1428e-5p
miR1429-3p
miR1429-5p
miR1430
miR159f
miR166e
miR169g
miR169j
miR1855
miR1862a
miR1862c
miR1863
miR1873
miR1884a
miR2091-5p
miR2094-5p
miR2096-5p
miR2098-5p
miR2100-5p
miR2102-3p
miR2102-5p
miR2104
miR2106
miR2118m
miR2118o
miR2123a
miR2862
miR2867-3p
miR2872
miR2880
miR2905
miR319a-3p
miR395f
miR395u
miR395v
miR395x
miR396e-3p
miR398a
miR399a
miR399b
miR399g
miR399l
miR408
miR5338
miR5340
miR5485
miR5488
miR5493
miR5496
miR5501
miR5504
miR5509
miR5510
miR5512
miR5514
miR5520
miR5524
miR5530
miR806a
miR806b
miR806c
miR806g
miR812g
miR812h
miR812i
miR812n-3p
miR818b
miR818c
miR818d
miR819b
miR819c-3p
miR819c-5p
miR819e-3p
miR819f
miR819h-3p
miR819h-5p
miR819j-3p
miR819j-5p
(b)
remained target genes
gained target genes
Percent of genes
1000
100
10
1
miR1317-3p
miR1317-5p
miR1319
miR1428b
miR1428d
miR1428e-
miR1429-3p
miR1429-5p
miR1430
miR166e
miR171g
miR1849
miR1861d
miR1861e
miR1861f
miR1861h
miR1861i
miR1861j
miR1862b
miR1866-3p
miR1877
miR1884a
miR2091-5p
miR2094-5p
miR2096-5p
miR2098-3p
miR2098-5p
miR2100-3p
miR2102-3p
miR2102-5p
miR2104
miR2118a
miR2118i
miR2118m
miR2121a
miR2123a
miR2124a
miR2862
miR2867-3p
miR2867-5p
miR2905
miR395c
miR395d
miR395e
miR395f
miR395g
miR395m
miR395q
miR395v
miR399a
miR399b
miR399i
miR408
miR5080
miR5081
miR5154
miR5159
miR5338
miR5485
miR5493
miR5496
miR5498
miR5508
miR5509
miR5514
miR5517
miR5520
miR5536
miR5538
miR5544
miR806a
miR806c
miR806g
miR809a
miR809c
miR809d
miR812a
miR812d
miR814c
miR815a
miR818b
miR818c
miR818d
miR819c-3p
miR819c-5p
miR819e-3p
miR819f
miR819h-3p
miR819j-5p
miR819k-3p
miR820c
